# FBS or BSA Inhibits EGCG Induced Cell Death through Covalent Binding and the Reduction of Intracellular ROS Production

**DOI:** 10.1155/2016/5013409

**Published:** 2016-10-18

**Authors:** Yin Zhang, Yu-Ying Xu, Wen-Jie Sun, Mo-Han Zhang, Yi-Fan Zheng, Han-Ming Shen, Jun Yang, Xin-Qiang Zhu

**Affiliations:** ^1^Department of Toxicology, Zhejiang University School of Public Health, Hangzhou, Zhejiang 310058, China; ^2^Department of Physiology, Yong Loo Lin School of Medicine, National University of Singapore, Singapore 117597; ^3^Department of Toxicology, School of Public Health, Hangzhou Normal University, 16 Xue Lin Street, Hangzhou, Zhejiang 310036, China; ^4^Collaborative Innovation Center for the Diagnosis and Treatment of Infectious Diseases, National Key Laboratory for the Diagnosis and Treatment of Infectious Diseases, The First Affiliated Hospital, Zhejiang University, Hangzhou, Zhejiang 310003, China

## Abstract

Previously we have shown that (−)-epigallocatechin gallate (EGCG) can induce nonapoptotic cell death in human hepatoma HepG_2_ cells only under serum-free condition. However, the underlying mechanism for serum in determining the cell fate remains to be answered. The effects of fetal bovine serum (FBS) and its major component bovine serum albumin (BSA) on EGCG-induced cell death were investigated in this study. It was found that BSA, just like FBS, can protect cells from EGCG-induced cell death in a dose-dependent manner. Detailed analysis revealed that both FBS and BSA inhibited generation of ROS to protect against toxicity of EGCG. Furthermore, EGCG was shown to bind to certain cellular proteins including caspase-3, PARP, and *α*-tubulin, but not LC3 nor *β*-actin, which formed EGCG-protein complexes that were inseparable by SDS-gel. On the other hand, addition of FBS or BSA to culture medium can block the binding of EGCG to these proteins.* In silico* docking analysis results suggested that BSA had a stronger affinity to EGCG than the other proteins. Taken together, these data indicated that the protective effect of FBS and BSA against EGCG-induced cell death could be due to (1) the decreased generation of ROS and (2) the competitive binding of BSA to EGCG.

## 1. Introduction

Green tea and green tea polyphenols, as naturally occurring antioxidants, have been associated with reduced risk for a number of human chronic and degenerative diseases including cancer [1]. The major green tea polyphenol (−)-epigallocatechin gallate (EGCG), which has a pyrogallol-type structure on the B-ring, can exert its actions by serving as an antioxidant or prooxidant [1, 2]. Interestingly, there is emerging evidence suggesting that the relevant mechanisms for the anticancer property of EGCG are not related to its antioxidative properties but rather are due to its prooxidative action and the direct interaction of EGCG with target molecules [2]. Through H-binding in 8 phenolic groups of EGCG, EGCG has been shown to bind with high affinity to multiple cellular proteins, including laminin receptor, the Bcl-2 homology 3 pocket of the antiapoptotic Bcl-2 protein, vimentin, and insulin-like growth factor I receptor [1]. It is believed that such direct interaction with cellular proteins affects many signaling pathways, which could lead to cell proliferation inhibition or even cell death, as well as the suppression of invasion, angiogenesis, and metastasis [1].

EGCG-induced cancer cell death is considered as one of the major events for its anticancer property; however, the underlying molecular mechanism remains to be fully elucidated. To date, results from most of the studies which examined EGCG-induced cell death suggested that caspase-dependent apoptosis was responsible [3–5], although nonapoptotic cell death was also reported in several studies [6, 7]. We have also investigated the cancer cell-killing effects of EGCG in a cell model, and interestingly, it was found that although EGCG induced cell death in both HepG_2_ and HeLa cells, it can only do so under serum-free condition [8]. Furthermore, we have also shown that the cells died of a nonapoptotic cell death via ROS-mediated lysosomal membrane permeabilization (LMP). However, why serum plays such an important role in deciding the cell fate remains to be answered.

Bovine serum, which contains a variety of plasma proteins, peptides, fats, carbohydrates, growth factors, hormones, inorganic substances, and so forth, is essential for the cells to grow* in vitro*. Bovine serum albumin (BSA) is the major component of bovine serum, and it has been shown that serum albumin can bind to EGCG through hydrophobic interactions or through H-binding [9]. Since the direct binding to cellular proteins has been suggested as a major mechanism for the toxic effects of EGCG, it is possible that in cell culture supplemented with serum EGCG would first bind to BSA or other components of the serum, which might interfere with/block the interaction of EGCG with cellular proteins. Consequently, this blockage could alleviate the toxic effects of EGCG. Therefore, in the current study, a series of experiments were conducted to prove this hypothesis. As reported here, FBS/BSA indeed can protect cells from EGCG-induced cell death by directly blocking the binding of EGCG to cellular proteins. In addition, the generation of ROS was also blocked by FBS/BSA.

## 2. Materials and Methods

### 2.1. Chemicals, Reagents, and Antibodies

(−)-Epigallocatechin-3-gallate (EGCG, >90%) was purchased from Zhejiang University Tea Research Institute (Hangzhou, Zhejiang, China). 5-(and-6)-Chloromethyl-2′,7′-dichlorodihydrfluoresceindiacetate acetyl ester (CM-H_2_DCFDA) and Hoechst 33342 were obtained from Invitrogen (Carlsbad, CA). Propidium iodide (PI) and other common chemicals were all purchased from Sigma-Aldrich (St. Louis, MO). The primary antibodies used in the study include the following: anti-poly (ADP-ribose) polymerase (PARP) and anti-caspase-3 (Cell Signaling, Danvers, MA); anti-*β*-actin, anti-*α*-tubulin, and anti-microtubule-associated protein 1 light chain 3 (LC3) (Sigma-Aldrich). The secondary antibodies, goat anti-rabbit IgG and rabbit anti-goat IgG, were all purchased from Thermo Scientific (Carlsbad, CA).

### 2.2. Cell Culture and Treatments

Human hepatoma cell line HepG_2_ was obtained from the American Type Culture Collection. Cells were grown in DMEM (Sigma-Aldrich) containing 10% fetal bovine serum (FBS, HyClone, Logan, Utah; the protein content for this specific lot is 3.5–5% (w/v)) and 1% penicillin-streptomycin (Invitrogen), in a 5% CO_2_ atmosphere at 37°C. “Serum-free medium” in this study referred to DMEM only without the addition of FBS.

### 2.3. Cell Viability Assays

Cell viability was quantified using PI exclusion test as previously described [8]. Briefly, cells were seeded into a 96-well plate at 5 × 10^3^ per well. 24 h later, cells were subjected to various treatments, followed by incubation with 10 *µ*g/mL Hoechst 33342 and 5 *µ*g/mL PI for 15 min at room temperature. For each sample, about 500 cells were visualized, randomly captured, and counted for cell viability based on the ratio of PI-positive/negative cells using an inverted fluorescent microscope (Nikon ECLIPSE TE2000-S, Japan).

### 2.4. Immunofluorescence Staining

In this study, *α*-tubulin was examined by immunofluorescence staining, based on an established method with modifications [10]. In short, treated cells were fixed with cold methanol (−20°C) for 10 min and permeabilized with 0.01% saponin in PBS for 10 min. After blocking with 1% BSA in PBS for 30 min, cells were incubated with anti-*α*-tubulin (mouse) primary antibody in a 1 : 500 dilution for 1-2 hrs at 4°C, followed by Alexa Fluor 488 goat anti-mouse secondary antibody (Invitrogen). The cells were examined using a confocal microscope (Olympus Fluoview FV1000), and representative cells were selected and photographed.

### 2.5. Western Blotting

The treated cells were lysed in whole cell lysis buffer (62.5 mM Tris-HCl at pH 6.8, 20% glycerol, 2% SDS, 2 mM DTT, 100 *µ*M PMSF, and proteinase inhibitor cocktail). Equal amounts of sample proteins (50 *μ*g) were subjected to SDS-PAGE and transferred to a polyvinylidene fluoride (PVDF) membrane (Bio-Rad, CA, USA). After blocking with 5% nonfat milk, the membrane was probed with designated primary and secondary antibodies and then developed with the enhanced chemiluminescence method (Thermo Scientific) and visualized with the Kodak Image Station 4000R (Kodak, Rochester, USA).

### 2.6. Detection of Reactive Oxygen Species

Analysis of intracellular ROS production was conducted as previously described [8]. Briefly, after various treatments, HepG_2_ cells were incubated with 10 *μ*M CM-H_2_DCFDA at 37°C for 15 min and analyzed under a fluorescence microscope (Nikon). Also, ROS generation was measured by microplate reader. In short, 10 mM CM-H_2_DCFDA stock solution (in methanol) was diluted 500-fold in PBS to yield a 20 *µ*M working solution. After various treatments, cells in each 96-well plate were washed twice with PBS and then incubated in 100 *µ*L working solution of CM-H_2_DCFDA at 37°C for 30 min. Fluorescence was determined at 485 nm excitation and 520 nm emission wavelength using an Infinite M200 microplate reader (Tecan, USA).

### 2.7. *In Silico* Study

The PDB structures of EGCG [11], BSA [12], PARP [13], caspase-3 [14], and LC3B [15] were available in the PDB databank (http://mgltools.scripps.edu/documentation/how-to/citing-pmv-adt-and-visi/). However, the PDB structure of tubulin (*homo spine*) was not available, so we used the tubulin structure of* Sus scrofa* instead [16]. The tubulin protein sequences of* homo spine* and* Sus scrofa* were compared by clusalX [17]. The Accelrys Discovery studio 4.5 program was used to construct the structure by removing other molecules from the original structure. Removing water molecules, adding hydrogen and PDBQT file of ligand, and molecule preparation were accomplished by using the AutoDock Tools 1.5 program.* In silico* docking analyses were performed using AutoDock Vina [18].

### 2.8. Statistical Analysis

The data were presented as mean ± SD from at least 3 independent experiments. Statistical analysis was calculated using Student's *t*-test (two-tailed distribution, unequal variance).

## 3. Results

### 3.1. BSA and FBS Inhibit EGCG-Induced Cytotoxicity

**Figure 1:**
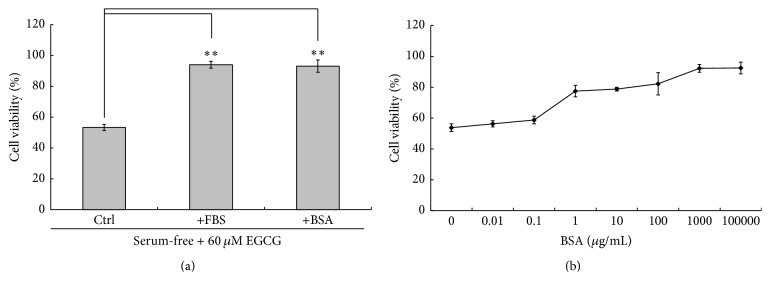
EGCG cytotoxicity was reduced by FBS/BSA. (a) HepG_2_ cells were cultured in serum-free medium or in the medium with 10% FBS or 10 mg/mL BSA for 1 hr and then treated along with 60 *μ*M EGCG for 24 hrs with the presence of BSA or FBS. The cell viability was determined by Hoechst-PI double staining (*n* = 3, mean ± SD). ^*∗∗*^ *p* < 0.001 comparing to the group without serum (Student's *t*-test, *n* = 3). (b) HepG_2_ cells were cultured in the medium with different concentrations of BSA for 1 hr and then treated along with 60 *μ*M EGCG for 24 hrs with the presence of BSA. The cell viability was determined by Hoechst-PI double staining (*n* = 3, mean ± SD).

Since previously we have shown that EGCG only induced HepG_2_ cell death under serum-free condition [8], it is believed that components of FBS should provide the protective effect against the toxicity of EGCG. Therefore, we first examined whether BSA, the major component of FBS, was responsible for such effect. HepG_2_ cells were cultured in serum-free medium or in the medium with 10% FBS or different concentrations of BSA for 1 hr and then treated with 60 *μ*M EGCG for 24 hrs. It was found that the cell viability was less than 60% for cells treated with EGCG in serum-free medium. However, in cells supplemented with 0.01 to 10,000 *μ*g/mL BSA, a concentration-dependent protective effect was clearly observed (Figure 1(b)). Furthermore, the addition of 10 mg/mL of BSA restored the cell viability to over 90%, almost the same as cells in medium with 10% FBS (Figure 1(a)), clearly demonstrating the protective effect of BSA against EGCG. Thus, it is concluded that BSA, the major component of FBS, is also a major contributor to the protective effect against EGCG-induced cell death.

### 3.2. BSA and FBS Inhibit EGCG-Induced ROS Generation

**Figure 2:**
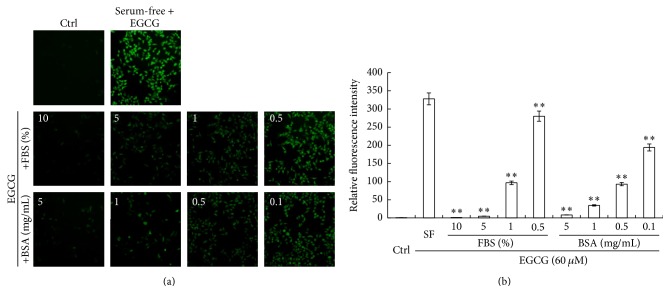
ROS production was reduced by FBS/BSA. HepG_2_ cells were cultured in serum-free medium or in the medium with different concentrations of FBS or BSA for 1 hr and then treated along with 60 *μ*M EGCG for 6 hrs with the presence of FBS or BSA. The intracellular ROS was detected by CM-H_2_DCFDA and analyzed under a fluorescence microscope. (a) Representative images showing intracellular ROS. (b) Quantitative analyses of intracellular ROS (*n* = 3, mean ± SD). ^*∗∗*^ *p* < 0.001, compared to the serum-free group.

HepG_2_ cells were cultured in serum-free medium or in the medium with different concentrations of FBS or BSA for 1 hr and then treated along with 60 *μ*M EGCG for 6 hrs. The intracellular ROS was detected by CM-H_2_DCFDA and analyzed under a fluorescence microscope. As we have reported, EGCG-induced cell death in serum-free medium was due to the generation of ROS [8]. On the other hand, the generation of ROS could be inhibited by adding FBS to the medium, as shown in Figure 2. Furthermore, a dose-dependent effect was observed for FBS. Similarly, adding BSA to the culture medium also inhibited the generation of ROS in a dose-dependent manner (Figure 2). Taken together, these data suggested that FBS prevented cells from the toxic effect of EGCG by inhibiting the generation of ROS and BSA played a major role in this function.

### 3.3. EGCG Forms Complex with Cellular Proteins

**Figure 3:**
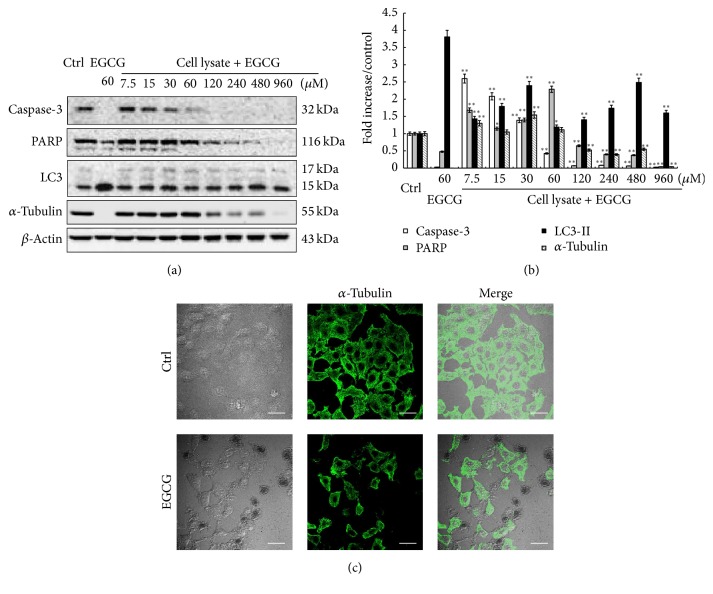
EGCG binds to cellular proteins. (a) Different concentrations of EGCG were added to cell lysates from untreated cells and incubated at 37°C for 9 hrs, then subject to Western blot. The cell lysate with no treatment was used as a control. The cell lysate from EGCG-treated cells (60 *μ*M, 9 hrs) was used as a positive control. A set of proteins, including caspase-3, PARP, and *α*-tubulin, became increasingly difficult to detect with increased EGCG. (b) Quantitative analyses of protein expression are presented as means ± SD (*n* = 3). ^*∗*^ *p* < 0.05, ^*∗∗*^ *p* < 0.001, compared to Ctrl group. (c) Immunofluorescence staining of *α*-tubulin was performed in HepG_2_ cells after EGCG (60 *µ*M) treatment for 9 hrs (scale bar: 20 *µ*m). EGCG exposure did not cause any significant changes in the expression level of *α*-tubulin.

It is known that EGCG can bind to many cellular proteins and form EGCG-proteins complexes. To determine whether such cellular protein-EGCG complexes can be formed, we first prepared HepG_2_ cell lysates and then incubated them with different concentrations of EGCG at 37°C for 9 hrs. To avoid protein degradation, the cell lysates were boiled and 2% SDS and protease inhibitor were added before adding EGCG. After incubation, a color change of cell lysates was observed, and when the lysates were separated on SDS-PAGE, some green materials remained in the gel pores (data not shown). More importantly, some cellular proteins, including caspase-3, PARP, and *α*-tubulin, became increasingly difficult to detect with increased EGCG (Figure 3(a)). For example, caspase-3 cannot be detected by 60 *μ*M or higher EGCG treatment, while PARP and *α*-tubulin became difficult to detect at 120 *μ*M or higher EGCG treatment. To prove that the decrease in these cellular proteins in SDS-PAGE was not caused by the decreased protein expression due to EGCG exposure, we examined the expression of *α*-tubulin in both control and EGCG-treated cells using immunostaining. As shown in Figure 3(c), EGCG exposure did not cause any significant changes in the expression level of *α*-tubulin, thus confirming that the failure to detect these proteins was not due to the decreased protein expression. In contrast, the detection of LC3 and *β*-actin was not affected, suggesting that there exists selective binding between EGCG and cellular proteins.

### 3.4. BSA Blocks the Complex Formation between EGCG and Cellular Proteins

**Figure 4:**
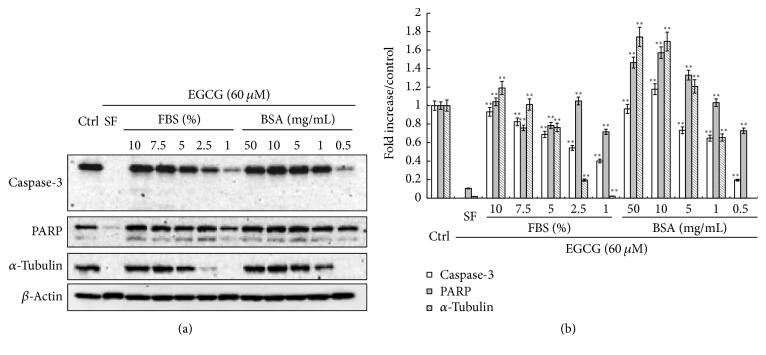
FBS/BSA blocks the binding of EGCG to intracellular proteins. HepG_2_ cells were cultured in serum-free medium or in the medium with different concentrations of FBS/BSA for 1 hr and then treated with 60 *μ*M EGCG for 9 hrs with the presence of FBS/BSA. Cell lysates were collected for Western blot. (a) The several proteins, including caspase-3, PARP, and *α*-tubulin, can be readily detected again in SDS-PAGE with increased concentrations of FBS/BSA in the culture medium. (b) Quantitative analyses of protein expression are presented as means ± SD (*n* = 3). ^*∗∗*^ *p* < 0.001, compared to the serum-free group.

Since EGCG has been reported to form complexes with BSA, we were wondering whether the protective effects of BSA may be associated with the competitive binding between BSA and cellular proteins to EGCG. HepG_2_ cells were cultured in serum-free medium or in the medium with different concentrations of FBS/BSA for 1 hr and then treated along with 60 *μ*M EGCG for 9 hrs. First it was observed that the color of cell lysates gradually returned to normal instead of green when increased concentrations of FBS or BSA were added back to the cell culture medium (data not shown). The several proteins, including caspase-3, PARP, and *α*-tubulin, can be readily detected again in SDS-PAGE with increased concentrations of FBS/BSA in the culture medium (Figure 4). For instance, caspase-3 and PARP can be detected when 1% of FBS or 0.5 mg/mL BSA was added to the cell culture, while higher concentration of FBS or BSA was required for the detection of *α*-tubulin. Taken together, these data supported our hypothesis that BSA and cellular proteins can competitively bind to EGCG.

### 3.5. *In Silico* Docking Analysis Reveals That BSA Has a Higher Affinity to EGCG

*In silico* docking analysis was conducted to evaluate the binding affinity of different proteins to EGCG. The results showed that BSA has three strong binding sites, with a maximum affinity of −10.4, −10, and −10.4 kcal/mol to EGCG, respectively; caspase-3 has two strong binding sites with a maximum affinity of −9 and −8.1 kcal/mol to EGCG, respectively; PARP, tubulin heterodimer, LC3A, and LC3B each has one binding site with a maximum affinity of −11.8, −10.5, −7.5, and −4.6 kcal/mol to EGCG, separately (Table 1). Based on the* in silico* docking analysis, it is concluded that BSA has a higher affinity than some cellular proteins to EGCG.

## 4. Discussion

To date, many studies have been conducted to evaluate the beneficial effects of EGCG, in particular, its antitumor properties. In most of the* in vitro* studies, serum was present in the cell culture systems. For example, 10% FBS was used in some of the studies [4, 5, 7, 19, 20], while 5% FBS was used in others [21, 22]. However, in our previous report, we have found that EGCG can only kill HepG_2_ or HeLa cells under serum-free condition [8]. Such difference could be due to the different types of cells used, the different concentrations of EGCG, or even the different sources of EGCG. Still, it should be kept in mind that FBS might play a key role in determining the toxicity of EGCG.

To understand why FBS can interfere with the toxic effects of EGCG on HepG_2_ cells, we conducted a series of experiments in an effort to elucidate the underlying molecular mechanism. First it was found that the EGCG-induced cytotoxicity was inhibited in a dose-dependent manner by the addition of FBS or BSA into culture medium. Next, it was revealed that FBS or BSA also inhibited ROS generation, a key step for EGCG-induced cytotoxicity, in a dose-dependent manner. The B-ring seems to be the principal site of antioxidant reactions and the antioxidant activity is further increased by the trihydroxyl structure in the D-ring (gallate) in EGCG [1]. EGCG can also be oxidized to generate ROS [2]. The mechanisms of the action of EGCG in cell culture systems, however, are complicated by the fact that it is not stable under most cell culture conditions.

The stability of EGCG can be affected by several factors, including the pH values of buffer solutions, oxygen concentration, temperature, metal ions, antioxidant concentration, and even the concentration of EGCG itself [23]. It has been reported that EGCG can form large water-soluble complexes with BSA or human serum albumin (HSA) and the complexes are stable to denaturation by detergents [24]. Under such experimental conditions, EGCG can form dimmers and epimers [25]. Some studies reported that the binding capacity of albumin contributed to the stabilization of EGCG and that albumin prevented EGCG oxidation by its antioxidant activity [26]. The possible mechanism for EGCG stabilization is that albumin directly prevented EGCG oxidation through a reversible interaction [27]. Therefore, in our study, it is reasonable to assume that by binding to EGCG BSA blocked the ability of EGCG to induce ROS generation (Figure 2).

The other major mechanism for the toxicity of EGCG has been attributed to its ability to bind to cellular proteins. In our previous work, we have noted an interesting phenomenon: the cell lysates from EGCG-treated cells in serum-free medium were green in color compared to the lysates from untreated cells. Furthermore, when such green cell lysates were analyzed using SDS-PAGE, it was found that they cannot enter the stacking gel totally and some visible green materials were left at the bottom of pores (data not shown). Meanwhile, it became difficult to detect a set of proteins in such cell lysates by Western blotting, such as caspase-3 and PARP [8]. Indeed, in our present study, we also found that EGCG can bind to some cellular proteins and formed complexes even under nonphysiological conditions, for example, incubation with cell lysate, which were inseparable by SDS-PAGE (Figure 3). Furthermore, such interaction between EGCG and cellular proteins also occurred under physiological conditions (Figure 4). Therefore, it is very possible that by binding to these cellular proteins, including those unidentified in this study, EGCG interfered with the normal functions of such proteins, which could eventually lead to cell death.

Interestingly, it should be noted that such binding is selective, indicating a structural preference of EGCG for certain proteins. In addition, BSA can block the binding of EGCG to these proteins in a dose-dependent manner (Figure 4). The interaction between EGCG and albumin has been well studied using a variety of techniques, such as gel electrophoresis, quartz crystal microbalance, fluorescence spectroscopy, Fourier transform infrared spectrometry, circular dichroism, and affinity capillary electrophoresis [27–30]. In this study, using* in silico* docking analysis, we provided a possible explanation for such effect of BSA: since BSA has a higher affinity for EGCG, it could competitively bind to EGCG first, thus blocking EGCG from binding to other cellular proteins. Indeed, such competitive binding has been observed between BSA and digitoxin for EGCG [24].

Taken together, in the current study, we answered the question why EGCG only showed toxic effects for HepG_2_ cells under serum-free condition. As it turned out, FBS/BSA blocked the generation of ROS induced by EGCG, the major intermediate responsible for the toxicity of EGCG. Furthermore, BSA could competitively bind to EGCG, thus blocking EGCG from binding to cellular proteins, another mechanism for the toxic effect of EGCG. Based on our results, it is clear that when evaluating the health benefits of EGCG using* in vitro* cell culture systems the presence/absence of FBS should be taken into consideration.

## Figures and Tables

**Table 1:** *In silico* docking analysis for the affinity of BSA and cellular proteins to EGCG.

Binding protein	Most stable structure	Strength (kcal/mol)
PARP	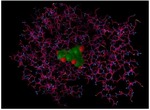	−11.8
Caspase-3	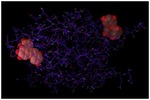	−9.0 −8.1
Heterodimer (*α*-tubulin and *β*-tubulin)	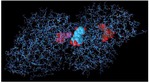	−11.6 −10.6 −10.5
BSA	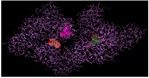	−10.2 −10.0 −10.4
LC3A	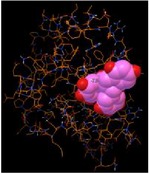	−7.5
LC3B	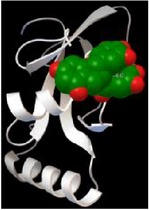	−4.6
